# Supplementary material for: Usefulness of the Thrombotic Microangiopathy Score as a Promising Prognostic Marker of Septic Shock for Patients in the Emergency Department
Source: J Clin Med. 2019 Jun 6;8(6):808. doi: 10.3390/jcm8060808 (PMC6617054; doi:10.3390/jcm8060808)

Supplementary Materials

Table S1. Univariate logistic regression analysis for predictors of 30-day mortality.

Variables	30-day Mortality	
	OR (95% CI)	P
Age (per 1 years)	1.028 (0.999–1.057)	0.056
Male (vs Female)	0.895 (0.439–1.828)	0.762
BMI (per 1 kg/m²)	1.031 (0.932–1.140)	0.555
SOFA score (per 1 point)	1.411 (1.258–1.584)	<0.001*
**Initial vital sign**		
Systolic blood pressure (per 1 mmHg)	0.995 (0.969–1.022)	0.722
Diastolic blood pressure (per 1 mmHg)	0.975 (0.932–1.020)	0.272
Heart rate (per 1 bpm)	1.003 (0.989–1.017)	0.643
Respiratory rate (per 1 bpm)	1.009 (0.923–1.104)	0.843
Body temperature (per 1 ^{o}C)	0.924 (0.886–0.965)	<0.001*
**Comorbidity**		
Hypertension	1.069 (0.519–2.202)	0.856
Diabetes mellitus	1.774 (0.875–3.600)	0.112
Cardiovascular disease	2.238 (1.030–4.861)	0.042*
Heart failure	1.006 (0.137–7.377)	0.995
Chronic kidney disease	0.518 (0.124–2.172)	0.369
Liver disease	1.145 (0.401–3.272)	0.801
Malignant	1.220 (0.562–2.650)	0.615
**Treatment**		
Admission to antibiotics time (per 1 hour)	0.837 (0.655–1.071)	0.158
Admission to vasopressor time (per 1 hour)	0.837 (0.651–1.076)	0.165
Antibiotics administration within 3 hours	1.831 (0.789–4.251)	0.159
**Laboratory data**		
White blood cell count (per 10^3/μL)	0.978 (0.937–1.021)	0.302
Hematocrit (per 1%)	0.969 (0.915–1.026)	0.279
Platelet count (per 10^3/μL)	0.998 (0.994–1.002)	0.315
Neutrophil count (per 10^3/μL)	0.972 (0.928–1.018)	0.235
Prothrombin time (per 1 INR)	1.582 (0.951–2.630)	0.077
Creatinine (per mg/dL)	1.145 (0.981–1.337)	0.086
C-reactive protein (per 1 mg/L)	1.001 (0.998–1.004)	0.437
Procalcitonin (per 1 ng/mL)	1.002 (0.992–1.012)	0.712
Albumin (g/dL)	0.347 (0.200–0.602)	<0.001*
Lactate (per 1 mmol/L)	1.268 (1.187–1.354)	<0.001*
Total CO2 (per 1 mmol/L)	0.809 (0.754–0.868)	<0.001*
Bacteremia	1.640 (0.796–3.378)	0.18
TMA score Time 0 (per 1 point)	1.856 (1.352–2.548)	<0.001*
TMA score Time 24 (per 1 point)	1.962 (1.403–2.744)	<0.001*

* $p < 0.05$; OR, odds ratio; CI, confidence interval; BMI, body mass index; SOFA, sequential organ failure assessment; TMA, thrombotic microangiopathy.

Table S2. Comparing scoring systems and biomarkers for the prediction of 30-day mortality using the area under the curve.

Variables	AUC (95% CI)	*p*	*p* (vs TMA Time 0)	*p* (vs TMA Time 24)	*p* (vs WBC)	*p* (vs CRP)	*p* (vs Procalcitonin)	*p* (vs Lactate)	*p* (vs SOFA)	*p* (vs APACHE2)
TMA Time 0	0.697 (0.589–0.789)	<0.001*	ref	0.34	0.191	0.033*	0.07	0.17	0.066	0.066
TMA Time 24	0.738 (0.600–0.853)	<0.001*	0.34	Ref	0.087	0.029*	0.049*	0.455	0.332	0.275
WBC count	0.591 (0.442–0.727)	0.213	0.191	0.087	Ref	0.648	0.68	0.012*	0.006*	0.014*
CRP	0.554 (0.493–0.644)	0.177	0.033*	0.029*	0.648	Ref	0.955	<0.001*	<0.001*	<0.001*
Procalcitonin	0.550 (0.427–0.669)	0.449	0.07	0.049*	0.68	0.955	Ref	0.001*	<0.001*	<0.001*
Lactate	0.800 (0.703–0.878)	<0.001*	0.17	0.455	0.012*	<0.001*	0.001*	Ref	0.968	0.689
SOFA	0.802 (0.727–0.865)	<0.001*	0.066	0.332	0.006*	<0.001*	<0.001*	0.968	Ref	0.689
APACHE2	0.822 (0.732–0.897)	<0.001*	0.066	0.275	0.014*	<0.001*	<0.001*	0.689	0.689	Ref

**p* < 0.05; AUC, area under the curve; CI, confidence interval; TMA, thrombotic microangiopathy; WBC, white blood cell; CRP, C-reactive protein; SOFA, sequential organ failure assessment; APACHE II, Acute Physiology and Chronic Health Evaluation.

Table S3. Comparison of the performance of the prediction of 30-day mortality with and without the TMA score by area under the receiver operating characteristic curve.

Prediction model	AUC (95% CI)	Difference of AUC (95% CI)	*p* (vs lactate)
Lactate	0.800 (0.703–0.878)	Reference	Reference
Lactate + TMA score Time 0	0.857 (0.785–0.919)	0.057 (0.012–0.117)	0.035*
Lactate + TMA score Time 24	0.892 (0.814–0.951)	0.092 (0.022–0.171)	0.018*

**p* < 0.05; AUC, area under the curve; CI, confidence interval; TMA, thrombotic microangiopathy.

Table S4. The TMA score as a predictor of 7-day mortality. Higher TMA score at admission (**A**) and 24 h (**B**) after admission were significantly associated with an increased risk of 7-day mortality among patients with septic shock.

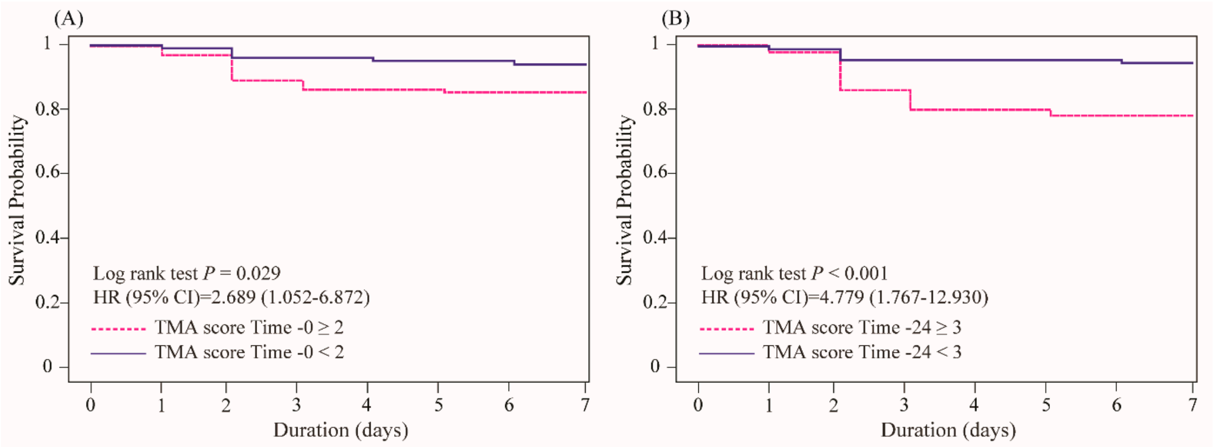

Supplement: Supplementary file 1 [file jcm-08-00808-s001.pdf]
